# Laboratory Genetic Testing in Clinical Practice

**DOI:** 10.1155/2013/532897

**Published:** 2013-07-14

**Authors:** Ozgur Cogulu, Yasemin Alanay, Gokce A. Toruner

**Affiliations:** ^1^Division of Genetics, Department of Pediatrics, Faculty of Medicine, Ege University, 35100 Izmir, Turkey; ^2^Department of Medical Genetics, Faculty of Medicine, Ege University, Izmir, 35100 Bornova, Turkey; ^3^Pediatric Genetics Unit, Department of Pediatrics, Acibadem University School of Medicine, 34457 Istanbul, Turkey; ^4^Cytogenetics & Molecular Genetics Laboratories, Institute of Genomic Medicine, UMDNJ-NJ Medical School, Newark, NJ 07109, USA

With the advances in genetic technology, the pages of the book of human biology and related diseases are turning over and over. Every new page is bringing a different vision into the life of humans not only limited to the scientific area but also to the daily life as well. The material used in genetic methods has found its place in this film with the discovery of a weak acid in the nuclei of white blood cells that we call DNA today. Unraveling the structure of DNA and chromosomes enabled us to find out the causes of many genetic diseases by cytogenetic and molecular genetic techniques. In situ hybridization with DNA probes followed these developments, and genome technology has advanced to the point of high-throughput sequencing nowadays. Throughout this period, the Human Genome Project provided a rapid increase of our insight into the field of human biology. The data obtained from this project allowed us to understand the underlying mechanisms of many genetic diseases and led to great interest among the scientific community.

On the other hand, the need for updated information in relation to the use of genetic techniques in the clinical practice of the clinicians increased in parallel with advances in genome technology. The practical use of the aforementioned genetic testing methods and proper interpretation of the generated test results have become a necessity not only for medical geneticists but also for the other specialists. Furthermore, physicians need to know how to order the most appropriate genetic test in the right time for the right indication to prevent under- or overutilization of those tests. That is why this special issue aimed to provide information about genetic techniques and their use in different research areas to improve knowledge, attitudes, and practices of physicians and researchers.

In this special issue, we would like to highlight both conventional and novel genetic methods, namely, classical cytogenetics, fluorescence in situ hybridization, PCR, RT-PCR, methylationspecific PCR, pyrosequencing, DNA sequencing, fully automated microarray-based assay, whole genome array CGH, MALDI-TOF mass spectrometry, and Southern blot analysis in a number of clinical conditions such as cancer, periodic fever syndromes, cardiomyopathy, myotonic dystrophy, retinal dystrophies, Wolf-Hirschhorn syndrome, and immunogenetic diseases.

Unquestionably those techniques do not comprise all of the methods in genomic studies; however they may give clues to understanding their potential use in clinical practice. Because genetic tests are unique and require robust quality assurance, quality assessment of genetic testing is undoubtedly necessary; therefore a study, which focused on the role of external quality assessments, was also featured in this issue as well. 

*Ozgur Cogulu* *Yasemin Alanay* *Gokce A. Toruner*

